# Outstanding Reviewers for *Chemical Science* in 2024

**DOI:** 10.1039/d5sc90122g

**Published:** 2025-06-19

## Abstract

We would like to take this opportunity to thank all of *Chemical Science*'s reviewers for helping to preserve quality and integrity in the chemical science literature. We would also like to highlight the Outstanding Reviewers for *Chemical Science* in 2024.

## Abstract 2

We would like to take this opportunity to highlight the Outstanding Reviewers for *Chemical Science* in 2024, as selected by the editorial team for their significant contribution to the journal.

## Graphical abstract

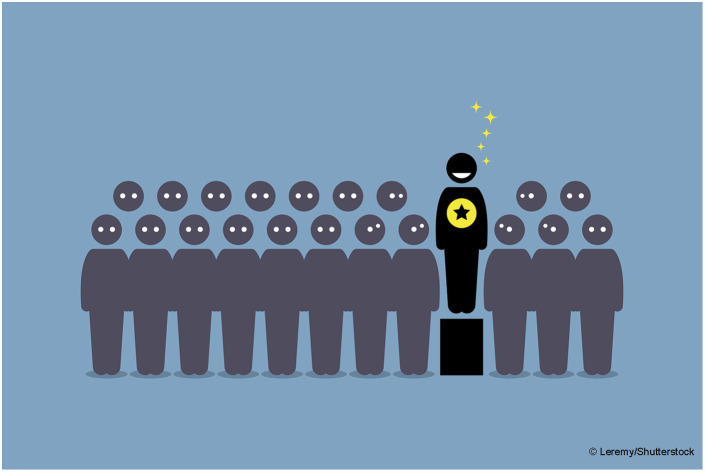

Each one of our outstanding peer reviewers has been carefully selected by our editorial team and includes active researchers who have made significant contributions to peer review and have gone above and beyond in their actions when reviewing for the journal during 2024.

As well as selecting reviewers who have provided a higher-than-average number of top-quality reviewer reports, we have also chosen to highlight those reviewers who have provided highly detailed reports and provided constructive feedback, as well as those adjudicative reviewers who have provided thoughtful and robust reports for manuscripts that have initially received conflicting recommendations.

In addition to our list of Outstanding Reviewers for *Chemical Science*, we feature our superb reviewer community year-round in our Reviewer Spotlight blog, which highlights a selection of reviewers every quarter. You can read our Reviewer Spotlight blog at https://blogs.rsc.org/sc/category/reviewer-spotlight/ and do keep an eye on our journal BlueSky (@chemicalscience.rsc.org), LinkedIn (Chemical Science Journal) and Facebook pages to see which reviewers have been particularly noteworthy.

2025 also marks the 15^th^ anniversary of *Chemical Science*, and we are celebrating the *Chemical Science* community with a dedicated Community collection. You will also find the research of many of our excellent reviewers highlighted as part of this special collection.

Our Outstanding Reviewers for *Chemical Science* in 2024 are:

Dr Joshua Barham

University of Regensburg

ORCID: 0000-0003-1675-9399

Professor Selvan Demir

Michigan State University

ORCID: 0000-0001-7983-9850

Dr Arundhati Deshmukh

Stanford University

ORCID: 0000-0002-7901-8814

Professor Abhishek Dey

Indian Association for the Cultivation of Science

ORCID: 0000-0002-9166-3349

Professor Amar Flood

Indiana University

ORCID: 0000-0002-2764-9155

Professor Ganna Gryn'ova

University of Birmingham

ORCID: 0000-0003-4229-939X

Professor Grace Han

Brandeis University

ORCID: 0000-0002-2918-1584

Professor David Harding

Suranaree University of Technology

ORCID: 0000-0001-8866-2401

Professor Laura Hartmann

Heinrich Heine University Düsseldorf

ORCID: 0000-0003-0115-6405

Professor Christian Heinis

École Polytechnique Fédérale de Lausanne (EPFL)

ORCID: 0000-0001-9982-9457

Professor Katja Heinze

Johannes Gutenberg-University

ORCID: 0000-0003-1483-4156

Professor Kevin Huang

University of South Carolina

ORCID: 0000-0002-1232-4593

Professor Hajime Kameo

Osaka Metropolitan University

ORCID: 0000-0002-6901-6372

Professor Kazuya Kikuchi

Osaka University

ORCID: 0000-0001-7103-1275

Dr Alexa Kuenstler

University of Illinois Urbana-Champaign

ORCID: 0000-0003-0432-2173

Dr Chidambar Kulkarni

IIT Bombay

ORCID: 0000-0001-8342-9256

Professor Jatish Kumar

IISER Tirupati

ORCID: 0000-0003-3078-6297

Professor Fei Li

Xi'an Jiaotong University

ORCID: 0000-0003-2081-8339

Dr John Mack

Rhodes University

ORCID: 0000-0002-1345-9262

Professor Mark MacLachlan

The University of British Columbia

ORCID: 0000-0002-3546-7132

Professor Biplab Maji

IISER Kolkata

ORCID: 0000-0001-5034-423X

Professor Vicent Moliner

Jaume I University

ORCID: 0000-0002-3665-3391

Professor Erin Stache

Princeton University

ORCID: 0000-0002-4670-9117

Professor Nathalie Steunou

Université de Versailles – Saint-Quentin-en-Yvelines

ORCID: 0000-0002-7049-7388

Dr William Unsworth

University of York

ORCID: 0000-0002-9169-5156

Professor Jianfang Wang

The Chinese University of Hong Kong

ORCID: 0000-0002-2467-8751

Professor Bing Yang

Jilin University

ORCID: 0000-0003-4827-0926

Professor Liliya Yatsunyk

Swarthmore College

ORCID: 0000-0003-3946-0939

We would also like to thank the *Chemical Science* Editorial Board and Advisory Board and the chemical sciences community for their continued support of the journal, as authors, reviewers and readers.

We continue to work on improving the diversity of our reviewer pool to reflect the diversity of the communities that we serve.

May Copsey, Executive Editor

